# Region-Dependent Responses to Oxygen–Glucose Deprivation and Melatonin in Neonatal Brain Organotypic Slices

**DOI:** 10.3390/antiox15010013

**Published:** 2025-12-22

**Authors:** Gorane Beldarrain, Unai Montejo, Marc Chillida, Jon Ander Alart, Antonia Álvarez, Daniel Alonso-Alconada

**Affiliations:** Department of Cell Biology and Histology, School of Medicine and Nursing, University of the Basque Country (UPV/EHU), 48940 Leioa, Spain

**Keywords:** neonatal hypoxia–ischemia, melatonin, oxygen–glucose deprivation, organotypic slice cultures

## Abstract

Hypoxic–ischemic encephalopathy remains a major cause of neonatal mortality and long-term neurological disability. Therapeutic hypothermia is currently the only available treatment in hospitals, but its efficacy is limited, making the search for alternative neuroprotective strategies essential. Melatonin has shown promising results in other models of hypoxia–ischemia, acting as a potent antioxidant and anti-inflammatory molecule. Here, we studied the effects of hypoxia–ischemia and melatonin treatment in two brain regions that are particularly vulnerable to hypoxic–ischemic injury. Neonatal rat organotypic slice cultures from the corticostriatal and hippocampal regions were subjected to oxygen–glucose deprivation and reperfusion (OGDR) and treated with melatonin (50 μM). Cell death (propidium iodide staining), redox state (GSH/GSSG ratio) and the inflammatory profile (Proteome Profiler) were analyzed. OGDR markedly increased cell death in both regions and melatonin treatment significantly reduced it. The GSH/GSSG ratio decreased only in the hippocampus after OGDR, but melatonin treatment elevated this ratio in both regions. In contrast, the inflammatory profile was more pronounced in the corticostriatal region, where the treatment strongly reduced proinflammatory mediators. These findings reveal region-specific mechanisms involved in the response to hypoxic–ischemic damage and support the potential of melatonin as a promising therapy for neonatal brain injury.

## 1. Introduction

Currently, 2–3 out of every 1000 term newborns experience some form of neurological dysfunction referred to as hypoxic–ischemic encephalopathy [1,2]. Approximately 24% of these infants die in neonatal intensive care units, and a significant number of survivors face serious long-term consequences [3]. These include cerebral palsy in 10–20% of cases, visual and hearing deficits in 40%, as well as motor and behavioral disorders such as epilepsy, global developmental delay, or autism [3].

Since therapeutic hypothermia was implemented in clinical practice, the risk of dying or developing such consequences has significantly reduced. However, several studies indicate that the benefits of hypothermia in neonates after hypoxia–ischemia (HI) might be limited [4,5,6], urging for new neuroprotective strategies. One of the reasons of a lack of an effective treatment is the complex physiopathology of hypoxic–ischemic encephalopathy, with concomitant processes encompassing oxidative stress and neuroinflammation, leading to cell death and brain tissue damage [7,8,9].

Melatonin (N-acetyl-5-methoxytryptamine) is a hormone synthesized in the pineal gland in response to light/dark cycles, so it is commonly used as a treatment for sleep disorders in children with few or no side effects [10]. It is also a potent antioxidant that scavenges highly toxic free radicals, increases antioxidant enzyme expression and inhibits nitric oxide synthase activity, the source of many free radicals that accumulate in cells after hypoxic–ischemic damage [11]. Several studies have demonstrated its neuroprotective potential, reporting reduced brain injury in rats in the short and long term [12,13], augmented neuroprotection when combined with hypothermia in piglets [14] or decreased cell death and inflammation in sheep [15], among others. However, much less is known about its region-specific impact within the brain. Most available studies addressing differences after melatonin treatment in different regions rely on either neuron cultures (which lack the architectural complexity of the brain), or in vivo studies (where the analyses are often restricted to histological outcomes). Organotypic brain cultures preserve the structural organization of the brain, including neurons, glia and their interactions. This provides an intermediate level of complexity that allows the study of molecular mechanisms such as oxidative stress and inflammation under controlled conditions across different brain regions. As reviewed by Humpel [16], organotypic slice cultures offer a more in vivo-like microenvironment than cell lines or dissociated primary cultures, while still allowing precise experimental control, reduced variability and a decrease in the number of animals required. Despite some limitations (they do not preserve the full neurovascular unit, systemic circulation or peripheral immune components), organotypic slice cultures have been widely used to efficiently study the effects of HI in the brain [17,18,19,20,21,22,23].

In neonates surviving intra-partum HI, the regional pattern of brain injury may change depending on various clinical factors, such as the gestational age or the severity of the insult [24,25]. Thus, different cerebral areas might not respond in the same way to the same insult, and a single treatment may not be able to offer neuroprotection in all brain regions.

Using organotypic brain slice cultures from neonatal rats, the aims of this work were (i) to compare the extent of the damage in corticostriatal and hippocampal regions by evaluating cell death, oxidative stress and inflammation and (ii) to determine whether melatonin differentially modulates these mechanisms across the two regions.

## 2. Materials and Methods

### 2.1. Organotypic Slice Cultures

All animal procedures were performed following the European Union regulations for animal research (Directive 86/609/EEC) and approved by the Animal Welfare Committee of the University of the Basque Country.

Organotypic slice cultures of the corticostriatal and hippocampal areas of the brain were prepared as previously described [22,26]. Briefly, brains were extracted from 6-day-old Sprague Dawley rats and the two hemispheres were separated. Coronal sections (350 μm thick) were obtained using a McIlwain Tissue Chopper (Ted Pella, Inc., Redding, CA, USA; Cat. No. 10180-220, 220V) and separated individually under a stereomicroscope. From these sections, those containing the corticostriatal region were selected and from adjacent sections, hippocampi were carefully dissected out. Both corticostriatal and hippocampal slices were then placed in Milicell culture inserts (Merck, Darmstadt, Germany; Cat. No. PICM0RG50) immersed in 1 mL of complete culture medium consisting of 50% MEM, 25% HBSS, 25% horse serum, supplemented with glucose (25 mM), B27 1X (Thermo Fisher Scientific, Waltham, MA, USA; Cat. No. 17504044), GlutaMAX 1X (Thermo Fisher Scientific, Waltham, MA, USA; Cat. No. 35050061), fungizone (Amphotericin B, 1.25 µg/mL; Thermo Fisher Scientific, Waltham, MA, USA; Cat. No. 15290018), penicillin–streptomycin (50 U/mL; Thermo Fisher Scientific, Waltham, MA, USA; Cat. No. 15070063), and gentamicin (50 µg/mL; Sigma-Aldrich, St. Louis, MO, USA; Cat. No. G1397). Slices of the same experimental group always originated from different rats to prevent intra-animal redundancy. Cultures were maintained in a 37 °C humidified incubator gasified with 5% CO_2_/95% O_2_ and medium was changed three times weekly for 14 days. Slices that showed altered morphology after 14 days in culture were discarded from the analysis.

### 2.2. Oxygen-Glucose Deprivation and Reperfusion (OGDR)

After 14 days in vitro, oxygen–glucose deprivation (OGD) was performed to simulate hypoxia–ischemia. Slices were placed in a hypoxic chamber (MIC-101, Billups-Rothenberg, San Diego, CA, USA) infused with a mixture of 95%N_2_/5% CO_2_ gas and the complete medium was changed to a glucose-free medium. Corticostriatal slices were maintained in such conditions for 1 h whereas hippocampal sections for 45 min. For the next 24 h, reperfusion was simulated by placing the cultures under normoxic conditions and replacing the medium with reperfusion medium, which had the same composition as the complete medium except for the horse serum. A shorter OGD duration was used for hippocampal slices to avoid extensive and irreversible hippocampal cell death due to its regional vulnerability, thus obtaining a similar level of injury in both regions that would still allow the detection of potential neuroprotective effects of melatonin. These different OGD times were also in line with previous works [18,23].

### 2.3. Preparation of Melatonin Treatment

Melatonin (Sigma-Aldrich, St. Louis, MO, USA; Cat. No. M5250), was dissolved in 0.05% DMSO and diluted in the reperfusion medium to a final concentration of 50 μM. The treatment was added immediately after the OGD process and maintained in the medium for 24 h. The final concentration of Melatonin was selected in accordance with previous studies demonstrating neuroprotective effects in organotypic and in vitro models of hypoxia–ischemia [13] and after dose–response experiments carried out by our group.

### 2.4. Experimental Groups

(1) Control: Slices maintained under normal culture conditions with complete medium and no treatment.
(2) OGDR: Slices subjected to oxygen–glucose deprivation followed by 24 h of reperfusion, to mimic HI and reperfusion-induced injury.
(3) OGDR + MEL: Slices subjected to oxygen–glucose deprivation and treated with 50 μM melatonin followed by 24 h of reperfusion to assess the potential protective effects of melatonin against HI and reperfusion-induced injury.

Experimental procedures, image analysis, and quantification were conducted in a blinded manner to ensure unbiased evaluation.

### 2.5. Cell Death Quantification

Dead cells were counted by staining the slices with propidium iodide (PI). PI is uptaken by dead cells as it is a polar molecule that can only enter the cell when the membrane is damaged. Once inside the cell, PI binds to DNA generating red fluorescence. PI (5 μg/mL) was added to the cultures after OGDR and maintained in the medium for 30 min. Slices were then observed using an inverted fluorescent microscope and the total number of PI positive cells (dead cells) were counted in situ at 10× (high power field, HPF) within the target region (hippocampus or corticostriatal area). Results are expressed as the ratio between the mean number of PI-positive/dead cells in the OGDR or OGDR+MEL groups and the control group (fold change).

### 2.6. Glutathione (GSH) and Oxidized Glutathione (GSSG) Quantification

To assess the redox state of the cells after OGDR and melatonin treatment, GSH and GSSG levels were measured using the Glutathione Fluorescent Detection Kit (Invitrogen, Waltham, MA, USA, Cat. No. EIAGSHF). For each experimental group, slices from a single culture were pooled to obtain sufficient tissue for analysis. The pooled samples were homogenized and processed according to the manufacturer’s instructions.

Briefly, we first obtained free GSH levels by measuring the fluorescence at 510 nm. The same samples were then incubated with a mix containing glutathione reductase and fluorescence was measured again to obtain total GSH levels. The GSSG levels were calculated dividing the difference between total GSH and free GSH by two:

GSSG=(totalGSH−freeGSH)÷2

The free GSH/GSSG ratio was calculated as an overall indicator of the redox state of the cell. Each measurement was performed in triplicate to ensure technical reproducibility. Data therefore represent technical replicates from a single pooled biological sample per condition and are presented as representative values.

### 2.7. Proteome Profile

The proteome profile of supernatants was assessed using a membrane-based sandwich immunoassay (Proteome Profiler Rat Cytokine Array Kit, R&D Systems, Minneapolis, MN, USA; Cat. No. ARY008). Briefly, supernatants of the same experimental groups were pooled, total protein was calculated using bicinchoninic acid assay and 400 μg of total protein from each pool were used to perform the assay. Each sample was mixed with a cocktail of biotinylated antibodies and incubated overnight in contact with a nitrocellulose membrane containing 29 different antibodies printed in duplicate. The next day, several washes were performed and streptavidin-HRP and the chemiluminescent reagent mix were added. The chemiluminescent signal was captured using a Syngene G:BOX Chemi HR16 (Syngene, Cambridge, UK) and pixel densitometry was obtained using Protein Array Tool (v2.0.0.1) [27] on MATLAB(R2024b, 24.2.0; The MathWorks, Inc., Natick, MA, USA), which turns the captured images into a list of relative protein levels.

### 2.8. Statistical Analysis

The normality of the figures was analyzed using the D’Agostino–Pearson test. For parametric data, a two-tailed, unpaired Student’s *t*-test was performed. In the same way, non-aparametric data were analyzed using the Mann–Whitney test. Statistical analysis was performed using the GraphPad Prism 10 software package (GraphPad Prism 10, GraphPad Software, San Diego, CA, USA) and data were considered significantly different if *p* < 0.05.

## 3. Results

### 3.1. Cell Death

In all experiments, PI uptake of the control group slices was considered as the basal level of cell death, so values of the other experimental groups were expressed as fold change vs. control.

**Figure 1:**
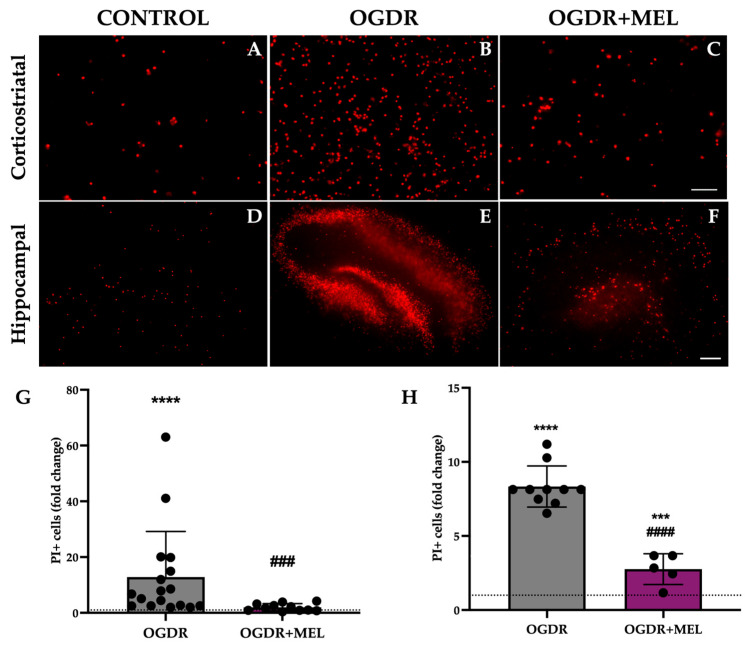
Cell death in corticostriatal and hippocampal slice cultures after OGDR and melatonin treatment. Cultures were subjected to 1 h (corticostriatal) or 45 min (hippocampal) of OGD and immediately treated with 50 μM melatonin followed by 24 h of reperfusion. Cell death was measured by PI uptake (**A**–**F**). Representative microphotographs of PI fluorescence in each of the experimental groups of the two regions ((**A**–**C**) Scale bar: 50 μm, (**D**–**F**) scale bar: 200 μm). (**G**,**H**). Graphs show PI+ cell counts in corticostriatal (**G**) and hippocampal (**H**) regions. n for control, OGDR and OGDR+MEL in the corticostriatal region was 16, 17 and 11 slices, respectively. n for control, OGDR and OGDR+MEL in the hippocampal region was 19, 10 and 5 slices, respectively. The dotted line at y = 1 represents the control values. *** *p* < 0.001 or **** *p* < 0.0001 vs. control group; ### *p* < 0.001 or #### *p* < 0.0001 vs. OGDR group.

In the corticostriatal region, slices that underwent OGDR showed significantly higher values of cell death compared to control, with the number of PI+ cells being 12 times higher in this group (*p* < 0.0001). Treating cells with melatonin after OGDR significantly reduced the number of PI+ cells observed in the OGDR group, as the fold change reduced from 12 to 1.9 (*p* < 0.001). Further, the treatment reduced the number of dead cells to control/basal levels, with no significant differences when comparing OGDR+MEL and control groups. Results are shown in Figure 1.

In the hippocampus, cell death was also significantly increased after OGDR, reaching values 8 times higher than those observed in the control slices (*p* < 0.0001). Again, melatonin treatment after OGDR significantly decreased the number of PI+ cells from a fold change of 8 to 2.75 (*p* < 0.0001). However, unlike in the corticostriatal region, the treatment did not reduce dead cells to control-like values in the hippocampus, as we found nearly 3 times more dead cells in the OGDR+MEL group compared to the control group (*p* < 0.001). Results are shown in Figure 1.

### 3.2. Oxidative Stress

To assess the redox state of the slices in the different experimental conditions, we measured the changes in the state of GSH and obtained the GSH/GSSG ratio, with decreased values as an indicator of cellular oxidative stress. These findings, obtained from pooled tissue samples analyzed in technical triplicates, should be interpreted as representative trends supporting the modulatory effect of melatonin on redox homeostasis.

**Figure 2:**
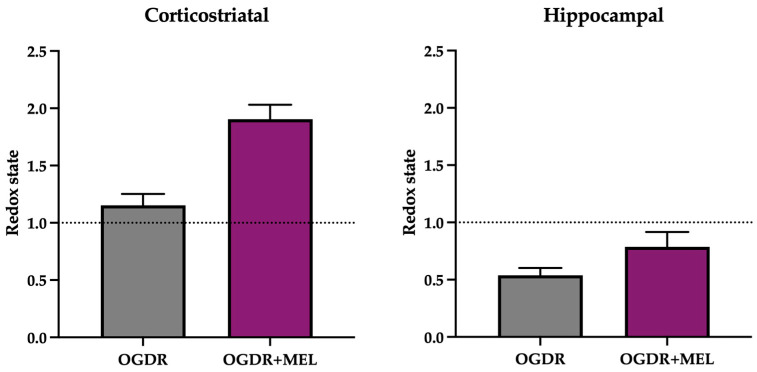
Redox state of the corticostriatal and hippocampal regions after OGDR and melatonin treatment. Cultures were subjected to 1 h (corticostriatal) or 45 min (hippocampal) of OGD and immediately treated with 50 μM melatonin followed by 24 h of reperfusion. GSH/GSSG ratio was calculated as an indicator of the redox state and is expressed relative to the control. n in the corticostriatal region was 6 slices in each experimental group. n in the hippocampal region was 9 slices in each experimental group. Slices were pooled and experiments were performed in triplicate. The dotted line at y = 1 represents the control values.

In the corticostriatal region (Figure 2), GSH/GSSG ratio did not change after OGDR compared to control samples. However, after treating the slices with melatonin, the GSH/GSSG ratio clearly increased, suggesting that melatonin modulates the cellular antioxidant system beyond its role as a direct antioxidant.

In the hippocampus (Figure 2), the redox state of the cells was markedly deteriorated following OGDR, as the GSH/GSSG ratio decreased to 0.53. After melatonin treatment, an improvement in redox status was found, as evidenced by a higher GSH/GSSG ratio in the OGDR+MEL group compared to OGDR. Nevertheless, this improvement did not reach control levels, thus suggesting that melatonin partially restores the cellular redox balance after OGDR.

### 3.3. Inflammatory Response

**Figure 3:**
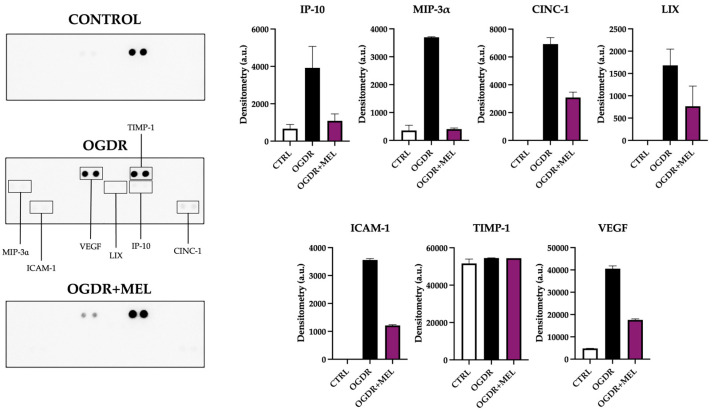
Proteome profile of the corticostriatal region after OGDR and melatonin treatment. Corticostriatal cultures were subjected to 1 h of OGD and immediately treated with 50 μM melatonin followed by 24 h of reperfusion. The proteome profile of supernatants was assessed using a membrane-based sandwich immunoassay (left side of the figure). The following were detected (graphs, right side of the figure): IP-10, MIP-3α, CINC-1, LIX, ICAM-1, TIMP-1 and VEGF. n was 6 slices in each experimental group. Samples were pooled and pixel densitometry was measured in duplicate.

Following OGDR, several mediators of the inflammatory response were altered in the corticostriatal region (Figure 3). IP-10 (Interferon-γ-induced Protein-10), MIP-3α (Macrophage Inflammatory Protein-3α), CINC-1 (Cytokine-Induced Neutrophil Chemoattractant-1) and LIX (Lipopolysaccharide-Induced CXC chemokine) are chemokines involved in leukocyte recruitment, which help amplify the inflammatory response. After OGDR, all four appeared upregulated compared to controls, where very little or none of these markers was detected. Remarkably, after treating the slices with melatonin, the levels of these chemokines were strongly reduced, recovering control levels as in the case of IP-10 and MIP-3α. The expression of ICAM-1 (Intercellular Adhesion Molecule-1), a molecule associated with cell adhesion and transmigration, was also affected: while no ICAM-1 was detected in the control samples, OGDR clearly upregulated its levels. Again, melatonin treatment notably reduced the expression of this inflammatory molecule.

Two markers related to tissue remodeling were also detected on corticostriatal samples. TIMP-1 (Tissue Inhibitor of Metalloproteinases-1), a tissue inhibitor of matrix metalloproteinases, showed elevated levels in all the experimental groups, with no differences between them. Compared to control samples, VEGF (Vascular Endothelial Growth Factor), a key regulator of angiogenesis and vascular remodeling, was strongly and mildly elevated after OGDR and OGDR+MEL, respectively.

**Figure 4:**
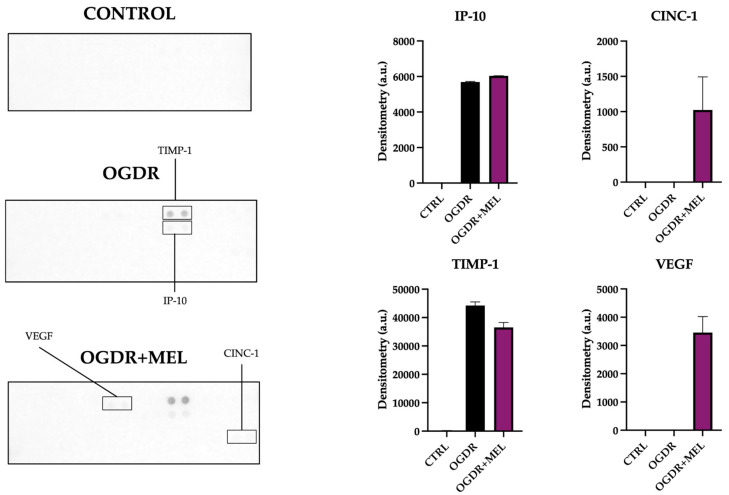
Proteome profile of the hippocampal region after OGDR and melatonin treatment. Hippocampal cultures were subjected to 45 min of OGD and immediately treated with 50 μM melatonin followed by 24 h of reperfusion. The proteome profile of supernatants was assessed using a membrane-based sandwich immunoassay (left side of the figure). The following were detected (graphs, right side of the figure): IP-10, CINC-1, TIMP-1 and VEGF. n was 10 slices in each experimental group. Samples were pooled and pixel densitometry was measured in duplicate.

In the hippocampus, the number of detected markers and their expression levels clearly varied compared to the corticostriatal region (Figure 4).

Regarding the recruitment phase of the inflammatory response, only IP-10 and CINC-1 were detected. The expression of IP-10 increased in OGDR slices compared to the control, where no IP-10 was detected. After treating cells with melatonin, the expression of this molecule did not differ from what was observed in OGDR slices. CINC-1, on the other hand, could not be detected in both the control and OGDR slices, but the supernatants of OGDR+MEL slices showed slightly increased levels of this marker. We did not detect MIP-3α, LIX or ICAM-1.

Tissue remodeling markers TIMP-1 and VEGF appeared again in hippocampi, but with a different profile compared to the corticostriatal region. Whereas TIMP-1 was not detected in control slices, its expression was strongly increased after OGDR and slightly reduced with melatonin. VEGF, unlike in the corticostriatal region, was only detected in the OGDR+MEL group, being absent in the non-treated OGDR group.

## 4. Discussion

Using organotypic brain slices from neonatal rats, we compared here the effects of HI and melatonin treatment in the corticostriatal and hippocampal regions, two brain areas particularly sensitive to asphyxia in the neonatal period. Our findings confirmed such sensitivity and similar extent in cell death but revealed inter-regional differences in oxidative stress and inflammation. Melatonin treatment was able to reduce cell death and oxidative stress in both brain areas, but its anti-inflammatory effects differed depending on the region.

When evaluating cell death, the degree of injury showed minor regional differences, with the corticostriatal area exhibiting slightly higher cell counts compared to the hippocampus. The latter is usually a more sensitive region to asphyxia [28], so this discrepancy may be attributed to the different oxygen-glucose deprivation durations used in this work (60 min for corticostriatal [23] and 45 min for hippocampus [18]), which were established to avoid irreversible cell death in the hippocampus. There are different factors that could be behind the regional differences observed in cell death. First, each region has different intrinsic properties, which are likely to contribute to different responses after damage. As observed in previous works, neurons and astrocytes from the cortex, hippocampus and striatum differ in the activity of superoxide dismutase and the expression levels of gluthatione or Bcl-xL, cellular components related to the regulation of the antioxidant and apoptotic responses [29]. Different cell types are also likely to have specific responses depending on the region, which is also probably behind the different patterns of injury. For example, astrocytes are usually more resistant than neurons to excitotoxic and oxidative stimuli, but in the striatum, astrocytes seem to be particularly vulnerable to OGD. A similar thing happens with cortical neurons, which are more sensitive than striatal and hippocampal neurons [29,30]. At the molecular level, brain regions can also differ in cell death pathways after HI, as CA1 neurons usually undergo caspase-dependent apoptosis [31], whereas cortical and striatal regions may show a combination of caspase-dependent apoptosis, necrosis and other forms of cell death [32,33].

As cell death usually correlates with (or is a consequence of) oxidative stress and inflammation [34], we next studied these parameters.

The corticostriatal region did not show major variations in oxidative stress (measured by the GSH/GSSG ratio), whereas data from the hippocampus revealed a significant drop in this ratio after OGDR, pointing to more severe oxidative damage in this region. These regional differences in oxidative stress may be due to several factors. Together with its high consumption of oxygen as one of the most metabolically active regions of the developing brain [35], the hippocampus contains a very high density of glutamatergic neurons compared to other regions [36], which makes this area especially vulnerable to excitotoxicity and the resulting overproduction of reactive oxygen species (ROS) [37,38]. Consistent with data from the present work, GSH levels of the CA1 pyramidal neurons significantly decreased after OGDR in slice cultures [39], leaving the hippocampus with a less robust antioxidant defense, especially during early development. For its part, the low impact of oxidative stress on the corticostriatal slices may be due to a greater redox capacity of the region or to a previously resolved oxidative peak, as inflammation was relevant in this area at 24 h after OGDR.

In the corticostriatal region, we observed increased values of IP-10, MIP-3α, CINC-1, LIX, ICAM-1 and VEGF. These results suggest a classical inflammatory cascade in this region, where leukocyte recruitment chemokines (IP-10, MIP-3α, CINC-1 and LIX) are predominant at the studied time point [40]. In corticostriatal primary cultures subjected to OGDR, Zhang and collaborators reported IP-10 was markedly increased, which contributed to neuronal injury [41]. Both CINC-1 and LIX are potent neutrophil-attracting signals that aggravate the outcomes of neurological damage [42,43]. Neutrophils contribute to the damage caused by ischemia by impairing vascular remodeling, worsening blood–brain barrier integrity, increasing microvascular occlusions and ultimately promoting neuronal death [44,45,46,47], which correlates with the cell death observed in our work after OGDR. Similarly to our data, Terao et al. also reported an increase in MIP-3α in a rat model of ischemia, which was associated with exacerbation of the injury [48]. Molecules involved in endothelial adhesion (ICAM-1) and tissue remodeling (VEGF) were also detected in the corticostriatal region, which suggests that although the region appears to be primarily at the leukocyte recruitment stage, the tissue might be initiating the next phases of inflammation, which involve vascular adaptation and structural reorganization [49,50]. Further, the induction of ICAM-1 in the cortex and the activation of VEGF after ischemia are well documented in rodents and both markers appear to be upregulated in response to damage [51,52]. To sum up, corticostriatal-augmented inflammation may amplify tissue injury, microvascular dysfunction and blood–brain barrier disruption [53,54,55].

In contrast, the hippocampal inflammatory profile was much milder, with only two markers (IP-10 and TIMP-1) upregulated at 24 h post-OGDR, both markers correlating with brain injury and long-term sequelae, as reported before [41,56].

These region-dependent differences in inflammation may be due to several factors, like the spatial heterogeneity of microglia and the temporal heterogeneity in cytokine kinetics, among others. Microglia plays a leading role in inflammation, and its density, morphology and gene expression is not uniform across all brain areas. Further, its diversity peaks at the developmental stage [57,58], so this may influence a region-specific response to damage. Another factor to consider is the different temporal window of inflammatory molecules depending on the region. In neonatal HI, canonical proinflammatory cytokines often increase within 3–6 h post-injury and return to baseline after 24 h in the hippocampus [59], while in the cortex or striatum, signals of these cytokines can be detected across the 12–48 h window post-insult [60].

Our work also studied the therapeutic capacity of melatonin in the two regions. Melatonin significantly reduced cell death in both corticostriatal and hippocampal areas, a neuroprotective effect in line with prior data using primary neuronal cultures [61,62], organotypic hippocampal slices [18,63] and in vivo HI models [32,33].

Melatonin also improved GSH/GSSG ratio, with similar increases in both brain areas. These data align with previous works reporting the antioxidant capacity of the molecule, enhancing the synthesis of GSH, as well as scavenging free radicals [64,65,66]. Here, OGDR especially affected the redox state of the hippocampus, so the observed redox rebalancing after the treatment could contribute to the observed decrease in hippocampal cell death [13,18,63].

As discussed previously, the main process behind damage of the corticostriatal region in our work appears to be inflammation. The inflammatory mediators upregulated after OGDR in this region (IP-10, MIP-3α, CINC-1, LIX, ICAM, and VEGF) were all reduced after melatonin treatment, thus confirming a potent anti-inflammatory effect of the treatment, described in other models [67].

A different response in inflammation was observed in the hippocampus after the treatment. The upregulated markers after OGDR (IP-10 and TIMP-1) were not significantly reduced and two new markers (CINC-1 and VEGF) were detected. Some of these changes might contribute to the reduction in cell death; for instance, the appearance of VEGF after the treatment might indicate tissue regeneration [68].

This study has some limitations. First, all measurements were obtained at a single time point (24 h post-OGD). This time-point corresponds to a window in which cell death, inflammation and oxidative stress are clearly detectable in our model, but it does not allow us to characterize their temporal dynamics. Earlier sampling could help define the onset of the oxidative and inflammatory responses, whereas later sampling would probably help clarify whether these processes persist or resolve in the longer term, leading to a more robust understanding of the evolution of deleterious and/or repairing mechanisms in the studied brain areas. While organotypic slices preserve local architecture and neuron–glia interactions, being a highly representative model for the study of new therapies in HI, they do not preserve the full neurovascular unit present in vivo. As commented before, OGD duration differed between regions to avoid irreversible cell death in the hippocampus, and slices had to be pooled for some analyses, which limits some statistical comparisons. Future work incorporating multi-timepoint sampling, study of the upstream processes behind the immune response and complementary in vivo validation will help refine the interpretation of these findings.

In summary, our work suggests that organotypic corticostriatal and hippocampal slices respond differently to OGDR, with inflammation playing a predominant role in the corticostriatal region and oxidative stress in the hippocampus. Melatonin treatment reduced cell death in both regions, targeting both harmful mechanisms. From a translational perspective, our findings reinforce the therapeutic potential of melatonin as a neuroprotective strategy in neonatal hypoxic–ischemic injury and highlight the importance of considering region-specific pathophysiology when designing and evaluating new treatments. Future studies should determine whether similar patterns of vulnerability occur in vivo and evaluate longer-term structural and functional outcomes. Such work will help bring our results a step closer to clinical application in neonates with hypoxic–ischemic encephalopathy.

## Data Availability

The original contributions presented in this study are included in the article. Further inquiries can be directed to the corresponding author.
